# Clinical diagnosis of the monogenic Ehlers-Danlos syndromes

**DOI:** 10.1515/medgen-2024-2060

**Published:** 2024-12-03

**Authors:** Fleur S. van Dijk, Chloe Angwin, Serwet Demirdas, Neeti Ghali, Johannes Zschocke

**Affiliations:** London North West University Health Care NHS Trust National EDS service, London North West University Health Care, NHS Trust Watford Road HA1 3UJ Harrow United Kingdom; London North West University Health Care NHS Trust National EDS service Watford Road HA1 3UJ Harrow United Kingdom; Erasmus Medical Centre Department of Clinical Genetics Dr. Molewaterplein 40 3015 Rotterdam Netherlands; Imperial College London Department of Metabolism, Digestion and Reproduction, Section of Genetics and Genomics SW7 2AZ London United Kingdom; Medical University Innsbruck Institute of Human Genetics Peter-Mayr-Str. 1 6020 Innsbruck Austria

**Keywords:** Clinical genetics, Ehlers-Danlos Syndromes, Extracellular matrix, Collagen

## Abstract

Monogenic Ehlers-Danlos syndromes (EDS) are a group of inherited connective tissue conditions that are clinically characterised by joint hypermobility, skin hyperextensibility and/or fragility, and generalised tissue fragility. Gene panel testing with massively parallel sequencing is currently gold standard to confirm diagnoses of the monogenic EDS types.

We aim to report on the (combination of) clinical features of the monogenic EDS types through text and photographs, to aid clinical diagnosis as despite the significant progress in genetic testing possibilities, a thorough clinical assessment which includes medical history, family history and physical examination remains important in the diagnostic process.

In addition, in those cases where no molecular diagnosis is possible, a clinical diagnosis can still guide management and surveillance.

## 1 Introduction

Ehlers-Danlos Syndrome (EDS) as a singular disorder was first reported in the beginning of the 20^th^ century by dermatologists Ehlers and Danlos who considered it a distinct clinical entity, characterised by skin hyperextensibility and fragility [1]**.** In the 1960s it became clear that there were in fact different types of EDS with different Mendelian inheritance patterns and both unique and overlapping clinical features. Detailed descriptions predominantly by rheumatologist Peter Beighton led to the 1986 Berlin nosology and subsequently the well-known Villefranche criteria, with Beighton first author of both publications. The Villefranche criteria divided EDS in 6 defined types with hypermobile EDS (type III in the Berlin nosology) thought to follow an autosomal dominant inheritance pattern [2].

This classification has since evolved, and it can be concluded that EDS now stands for “Ehlers-Danlos syndrome**s**” (plural) encompassing 13 different types [3]. Whilst hypermobile EDS is a clinical diagnosis with strict diagnostic criteria since 2017, the other 12 EDS types are proven monogenic connective tissue conditions that generally share clinical features of joint hypermobility, skin hyperextensibility and/or fragility, and generalised tissue fragility. A timely diagnosis of an EDS type is important to ensure targeted management and surveillance in affected individuals. It is therefore best practice to refrain from the general term “EDS” and instead define which type of EDS it concerns [4]. Unfortunately, both affected individuals and health care professionals often still use the general term “EDS” as a disease entity, which can lead to confusion and consequently can delay appropriate management. This can be harmful especially for individuals with monogenic types of EDS at risk of significant complications.

The underlying genetic causes of monogenic EDS types (except for periodontal EDS) can be grouped in genes encoding Collagen type I, III, V and XII, enzymes involved in their biosynthesis, or enzymes involved in proteoglycan biosynthesis [3]. Gene panel testing by massively parallel sequencing is the current gold standard to confirm monogenic diagnoses and this also applies to the EDS types. Despite the significant progress in genetic testing possibilities, a thorough clinical assessment including medical history, family history and physical examination, remains important in the diagnostic process. Dependent on level of clinical expertise in EDS, this can lead to a clinical diagnosis of monogenic EDS although this still requires confirmatory genetic testing. Clinical features or phenotype, applied at different strengths, contribute to the ACMG classification criteria [5] assigning identified genetic variant(s) as (likely) benign, variant of unknown significance (VUS) and (likely) pathogenic. In the absence of a reliable molecular diagnosis – which may be due to non-identification of variant(s) in non-coding regions, structural variants, or a VUS – a clinical diagnosis can still give clarity, aid management and direct further investigations.

Since 2017, the 12 monogenic types of Ehlers-Danlos syndromes each have their own major and minor clinical criteria, which were defined through an international consortium [6]. We aim to report on the (combination of) clinical features of the monogenic EDS types through text and photographs, to aid clinical diagnosis. Where possible we have also made references to open access case reports, original articles, and reviews, so that further detailed information can be easily accessed. All minor criteria can be found in the open access paper by Malfait et al. 2017 [5]. An overview of gene-specific minor criteria is available in **supplementary** **table 1.**

## 2 Major criteria for diagnosis of the 12 monogenic EDS types

Below follows a clinical description of the major criteria for diagnosis of the molecularly defined types of EDS [6], where indicated with additional information regarding management and surveillance. The prevalence of the different monogenic EDS types is based on the proportion of a particular population found to be affected at a specific time and is taken from available literature. It is suspected that historical prevalence for many of the rare EDS types may be an underestimate due to, for example, increased availability of genetic testing worldwide resulting in more diagnoses in individuals with milder clinical features. Additionally, the prevalence of recessive EDS types may be increased in populations with a higher frequency of consanguinity.

### Classical EDS (cEDS, prevalence 1;20,000, (7) autosomal dominant, mostly variants in *COL5A1* or *COL5A2*, rarely specific variants in *COL1A1*)

The major criteria of classical EDS are (i) generalised joint hypermobility and (ii) skin hyperextensibility and atrophic scarring. Drivers of the diagnosis in childhood are often the tendency to bruise easily, as well as skin fragility leading to splitting of the skin where there is a skin laceration extending through the epidermis and dermis. Subsequent impaired wound healing results in atrophic scars. These are characterised by thin, wrinkly scar tissue, hence the old term “cigarette paper or papyraceous scarring”. Atrophic scarring is often most noticeable on forehead, knees and shins and can occur even after mild trauma for example with parents reporting their child falling on a soft carpet leading to splitting of the skin and subsequent impaired wound healing with atrophic scarring. Individuals, particularly in families with multiple affected family members, will often manage their own wounds with adhesive tapes (steri-strips) and only when strictly necessary attend the A&E to seek medical assistance for sutures given the increased frequency of injuries. Occasionally, the extent of skin injury requires skin grafting. In case of deep skin wounds, double layered stitching is recommended [8]. Interestingly, surgical incisions often heal well without atrophic scarring. For painful bruising with swelling often Tranexamic acid is advised to have at home for when needed. This is applicable to all individuals with rare EDS types who are prone to painful bruising with swelling. It is often reported by adults that the frequency of accidents leading to skin splitting is high from toddlerhood and decreases from young adulthood. Many mention that being cautious is the main reason for this along with a general reduction in physically boisterous activities.

In young children, the hyperextensibility of the skin can easily be missed, as the skin is also still very elastic and needs to be pulled with some force before it becomes clear how far it can stretch. Testing skin hyperextensibility at stretched elbows and other areas of the body for example dorsum of the hand and neck, often gives a clear indication about presence of hyperextensibility.

In childhood, generalised hypermobility with or without (sub)luxations and joint pains are often noted. Adults tend to often develop joint pains due to osteoarthrosis starting from as early as thirties. They often consider this their main problem and therefore specialist rheumatology and physiotherapy input can be very valuable.

Further data from larger cohort studies should provide more clarity regarding frequency of cardiovascular complications including arterial aneurysms/dissections which so far are considered rare [9]. Importantly, several heterozygous variants causing Glutamic acid>Lysine changes in *COL3A1* have been reported to cause clinical features of classical EDS with a hyperextensible skin and skin fragility but also generalised tissue fragility as seen in vascular EDS [10].

### Vascular EDS (vEDS, prevalence 1;50,000 [11], autosomal dominant, mostly variants in *COL3A1,* rarely specific Arg>Cys variants in *COL1A1*)

Vascular EDS is one of the most well-known types of EDS due to presence of generalised tissue fragility. Major criteria include (i) arterial aneurysm and/or dissection, (ii) sigmoid colon perforation in absence of diverticular disease or other colon pathology, (iii) uterine rupture during the third trimester in the absence of previous C-section and/or severe peripartum perineum tears and (iv) carotid-cavernous sinus fistula (CCSF) formation in the absence of trauma. Referrals for assessment for vEDS can therefore come from a variety of different specialties including Haematology, Rheumatology, Vascular surgery, Cardiology, Respiratory medicine, Intensive care medicine, and Paediatrics.

There are multiple minor diagnostic criteria, most typically the combination of easy and/or spontaneous bruising, bruising at unusual locations, and translucent thin skin with visible veins. However, it needs to be emphasized that there are individuals who do not have clear features of vEDS on physical examination and nevertheless have generalised tissue fragility. Therefore, vEDS remains a diagnosis that without positive family history can sometimes be only made after a (major) complication in the proband has occurred. Also, it is important to emphasize that the intrafamilial and interfamilial variability in this condition is striking regarding clinical features on physical examination, (age at) occurrence of clinical events, and type of clinical events. Importantly, management through an expert service has been shown to have a favourable effect on survival [12, 13]. Management consists of lifestyle advice including seeking support in acute situations, recommending aerobic exercise whilst still being able to hold a conversation, and advising against isometric exercise. An emergency card with explanation about vEDS for medical health care professionals should be provided. Pharmacological therapy includes blood pressure lowering medication such as Celiprolol [12] or combined therapy with beta blocker and angiotensin II receptor blocker [13]. Regular cardiovascular imaging is recommended, and it is important to advise on conservative management above invasive or endovascular procedures unless unavoidable [4, 14]. Due to the risk of death during pregnancy of 5.3 % in women with vEDS [15], obstetric management should take place in tertiary obstetric centres [13]. International guidance regarding pregnancy management in vEDS has been published through the ERN network VASCERN (https://vascern.eu/).

### Kyphoscoliotic EDS, Prevalence 1:100,000, Biallelic variants in* PLOD1* or *FKBP14*.

Kyphoscoliotic EDS is a well-known type of EDS and *PLOD1* variants were the first reported recessive cause of a monogenic EDS type. Major criteria include (i) congenital muscle hypotonia, (ii) congenital or early onset kyphoscoliosis (progressive or nonprogressive), and (iii) GJH with dislocations/subluxations. Rupture of medium-sized arteries has been reported as part of the minor criteria, and therefore cardiovascular surveillance should be available to affected individuals [16].

The skin features in *PLOD1-*kEDS are similar to those seen in cEDS, with presence of skin fragility, atrophic scarring and easy bruisable skin which are part of the gene-specific minor criteria. There are reports of individuals without congenital or early onset kyphoscoliosis in whom the initial clinical diagnosis was thought to be cEDS [16]. A minority of individuals with *PLOD1* kEDS was reported to have scleral rupture after minimal trauma [17]. Eye protection is recommended to avoid accidental damage during physical activities.

*FKBP14-*kEDS is different in that skin fragility is often absent, and individuals often have widespread follicular hyperkeratosis as well as congenital hearing impairment (sensorineural, conductive, or mixed) [18].

### Arthrochalasia EDS, Prevalence < 1:1,000,000, autosomal dominant, variants in *COL1A1* or *COL1A2* that lead to complete or partial deletion of exon 6

The clinical hallmark of aEDS is (i) congenital bilateral hip dislocation in conjuction with (ii) severe generalised joint hypermobility and (sub)luxations [19, 20]. There is a genotype-phenotype relationship in that musculoskeletal features, including liability to fractures, tend to be more severe in individuals with *COL1A1* variants. Access to musculoskeletal services can address their needs, and the condition is often well managed through services that also have experience with osteogenesis imperfecta (OI) which is the most common manifestation of heterozygous pathogenic variants in *COL1A1* or *COL1A2* [19]. People with aEDS can have skin features as seen in individuals with cEDS, including hyperextensible skin which is the third major criterium (iii).

### Dermatosparaxis EDS (dEDS, prevalence <1:1,000,000, biallelic variants in *ADAMTS2*

With only 15 individuals reported in childhood – with an age range from birth to 16 years – and one patient being followed-up to adulthood (19 years), the clinical criteria for dEDS are extensive comprising 9 major criteria. In the authors opinion the most important features are (i) extreme skin fragility with congenital or postnatal skin tears, (ii) characteristic craniofacial features as seen in figure 2D (iii), redundant almost lax skin with excess folds at the wrists and ankles, and (iv) severe bruisability with a risk of subcutaneous hematomas and haemorrhage. Tissue fragility has been noted in some individuals with bladder rupture likely due to bladder diverticulae, and diaphragmatic rupture following vomiting. In adulthood one individual also developed diaphragmatic rupture with hiatus hernia (###, personal communication).

### Brittle Cornea Syndrome, biallelic variants in *ZNF469* or *PRDM5*

As the name suggests, all the major features of individuals with Brittle Cornea Syndrome (BCS) relate to the eye, and particularly the cornea. They include (i) thin cornea with or without rupture, (ii) early onset, progressive keratoconus or(iii) keratoglobus and (iv) blue sclerae. The latter can of course be identified on physical examination, taking into consideration that blue sclerae in the first 12 months can still be physiological and can also be seen in individuals with other inherited tissue conditions. Mixed hearing loss can also be present, as well as musculoskeletal features such as developmental dysplasia of the hip, scoliosis, and joint hypermobility. Dhooge et al point out that individuals who do not present with symptoms related to corneal fragility are at risk of diagnostic delay and emphasize the importance of identifying features of an inherited connective tissue condition. This is a reason for inclusion of the BCS-related genes in inherited connective tissue disorder gene panels [21].

### Cardiac valvular EDS, biallelic loss of function variants in *COL1A2*

Cardiac-valvular EDS is a very rare EDS type as so far only 7 individuals with an age range from 6–65 years have been reported and is caused by bi-allelic loss of function (LOF) *COL1A2* variants leading to mRNA decay. The main criteria are (i) severe progressive cardiac-valvular problems of mitral and aortic valve. On physical examination (ii) distal or generalised joint hypermobility is often noted as well as (iii) skin involvement with hyperextensibility, thin skin with visible veins, atrophic scarring, and easy bruising. Since then, Guarnieri et al. [22] reported on 5 previously and 2 newly diagnosed patients. They noted that the core cvEDS phenoype consists of joint hypermobility limited to, or more marked at, hands and feet (100 %), skin hyperextensibility (100 %), mitral valve insufficiency (83 %), genua recurvata (66 %), bilateral pes planus (66 %), soft/doughy/thin skin (66 %), variable atrophic scarring (50 %), easy bruising and bilateral inguinal hernia (50 %). Of course, as these percentages are based on occurrence of clinical features in a very small group of affected individuals, the description of the core cvEDS phenotype could change significantly depending on additional reported individuals with cvEDS.

### Classical-like EDS (clEDS), prevalence < 1:1,000,000, autosomal recessive *TNXB-*clEDS & autosomal recessive AEBP1 -clEDS

In the 2017 classification, clEDS was exclusively linked to pathogenic variants in *TNXB*; after the identification of *AEBP1*- clEDS, the condition is also denoted as clEDS type 1. Affected individuals typically have a (i) hyperextensible skin without atrophic scarring and (ii) generalised joint hypermobility with or without dislocations. They often bruise very easily and are prone to haematomas (iii). Foot abnormalities are very common (81 %) including broad/plump forefoot, brachydactyly with excessive skin, pes planus, hallux valgus, and painful soles of the feet. The most specific foot abnormality was broad feet with brachydactyly. Importantly, tissue fragility has been reported including gastrointestinal fragility with oesophageal, small bowel and/or large bowel ruptures (16 %), vascular fragility leading to major events (5 %) and other fragility including tracheal rupture after intubation and defect of nasal cartilage after nose blowing (4 %). Therefore, invasive procedures are advised against unless unavoidable and should then be executed with caution and awareness of potential complications due to tissue fragility. A baseline echocardiogram of the heart is currently recommended [23–26].

### *AEBP1-* clEDS

No specific diagnostic criteria have yet been formulated for *AEBP1*-EDS, also called clEDS type 2. As the name suggests, individuals with this condition share features with people with cEDS and TNXB clEDS. All individuals reported so far have GJH, hyperextensible skin, easy bruising and in the majority also atrophic scarring. Intriguingly, thinning of hair was present in 6/11 (55 %) of individuals reported by Angwin et al. [27]. A recent overview of 15 individuals with *AEBP1* clEDS [28] also referencing Yamaguchi et al. [29] reported on 3/15 individuals (20 %) with arterial aneurysm/dissection, 2/15 (13 %) with aortic root dilatation highlighting the importance of cardiovascular surveillance, and 2/15 (13 %) with bowel rupture suggesting avoidance of invasive procedures unless absolutely necessary.

### Myopathic EDS, prevalence < 1:1,000,000, heterozygous or biallelic variants in *COL12A1*

To date, 25 individuals with myopathic EDS (mEDS) from 16 families have been reported, with autosomal recessive inheritance in two families [30]. Individuals with myopathic EDS share features with Bethlem and Ullrich congenital myopathies. mEDS is clinically characterised by (i) congenital muscle hypotonia, and/or muscle atrophy, that intriguingly improves with age. In addition, (ii) proximal joint contractures (knee, hip, and elbow) can be present, as well as (iii) hypermobility of distal joints. Atrophic scarring and soft, doughy skin may be observed. As there are only few individuals reported so far, the clinical criteria are likely to become more specific with time. It can be argued that because of its strong myopathic component mEDS should be classified as a congenital myopathy rather than a rare EDS type.

### Periodontal EDS, prevalence < 1:1,000,000, specific gain-of-function variants in *C1R* or *C1S*

Of all the different rare EDS types, pEDS is certainly the most intriguing one regarding the underlying pathogenic mechanism, which involves components of the classical pathway of the complement system. It is ultimately caused by activation of the C1r complement subunit which has collagenase function and may also lead to incomplete activation of the complement system. A pathognomonic feature in people with pEDS is (i) lack of attached gingiva which is the keratinised gingiva that connects the gingiva to the dental bone [31]. Individuals typically experience (ii) gingivitis and aggressive periodontitis leading to periodontal bone loss at a very young age with complete dental loss at an average age of 20 years. The diagnosis of lack of attached gingiva requires the very trained eye of a periodontal expert and may be easily missed. However, the history of receding gums with teeth becoming loose and falling out in combination with gum recession and (ii) presence of pretibial plaques – often symmetrical brownish discoloured plaques on the shins that can also involve the calves – are very suggestive of pEDS. In addition, imaging studies in adults with pEDS have revealed white matter abnormalities in almost all adults who underwent imaging studies; there are usually no clear neurological symptoms although symptoms such as migraines and poor memory have been recorded. Some individuals develop a hoarse or high-pitched voice. Rare complications include aneurysms/dissections and organ rupture, and cardiovascular surveillance is advised [32–33]. Excellent dental hygiene is recommended with electric toothbrushes, interdental brushes, and 3-monthly professional dental cleaning. This will limit formation of dental plaque and may reduce subsequent excessive C1r/C1s activation that leads to severe periodontitis and loss of periodontal bone and consequently teeth [31–32]. However, whilst this may reduce the degree of the inflammatory response, the underlying activation remains.

### Musculocontractural EDS, prevalence < 1;1,000,000, biallelic variants in *CHST14* and *DSE*

Individuals with mcEDS are usually diagnosed in childhood as they have multiple congenital contractures specifically adduction-flexion contractures and/or talipes equinovarus in combination with specific craniofacial features and characteristic skin features including skin hyperextensibility, easy bruisability, skin fragility with atrophic scars, increased palmar wrinkling. Individuals with mcEDS can develop multiple complications of which some are also part of the minor criteria. An international review of 66 individuals with recessive *CHST14* variants was published in 2022 [34] as well as review on 14 individuals with mcEDS due recessive *DSE* variants [35] which should guide revision of gene specific criteria formulated in 2017.

### Spondylodysplastic EDS, prevalence <1:1,000,000, biallelic variants in *B3GALT6*, *B4GALT7* and *SCL39A13*

Individuals with spondylodysplastic EDS are also often diagnosed in childhood due to major features of (i) short stature, (ii) muscle hypotonia (ranging from severe congenital, to mild later-onset) and (iii) bowing of limbs leading to a suspicion on a skeletal dysplasia. The associated cutaneous skin features are important in the diagnostic process and likely the reason why this condition is classified as a monogenic EDS type. These features consist of skin hyperextensibility, doughy skin and thin translucent skin. As there are three different genetic causes that lead to spEDS there are gene-specific criteria as well.

### OIEDS1, OIEDS2 and COL1-related overlap disorder condition

In 2020 Morlino et al. [36] coined the term COL1 overlap disorder pertaining to individuals with previously reported OIEDS1 and OIEDS2 due to combined symptoms of osteogenesis imperfecta type 1 (non-deforming OI with blue sclerae) and Ehlers Danlos syndromes due to structural *COL1A1* and *COL1A2* variants without fulfilling the clinical criteria for these conditions. Major criteria for COL1-related overlap disorder were defined as (i) blue sclerae, (ii) flatfoot with valgus deformation of the hindfoot, (iii) generalised joint hypermobility according to age, (iv) significantly soft and doughy, and/or hyperextensible skin. Minor criteria consisted of (i)dolichostenomelia in adults, (ii) hearing loss, (ii) short stature, (iv) two or more atrophic scars, (v) two or more fractures in pre-pubertal age, (vi) two or more joint dislocations, (vii) two or more fractures injuries and/or ruptures of ligaments, tendons and/or muscles. Genetic testing of *COL1A1* and *COL1A2* genes was encouraged in presence of ≥3 major criteria or one major and ≥ 4 minor criteria.

Venable et al. [37] subsequently reported on a cohort of 34 individuals with (likely) pathogenic variants in *COL1A1*/*2* with an initial diagnosis of OI, unspecified EDS, and interestingly, hypermobile EDS. They suggested that features such as easy bruising, vascular fragility, and striae as well as positive family history could be incorporated in the COL1-related overlap disorder criteria. They also argued for a comprehensive checklist to document full fracture history, dental history, wound healing abnormalities, physical exam with Beighton scoring, sclerae colour, and texture/extensibility of the skin, essentially describing the clinical assessment by a specialist in inherited connective tissue conditions.

**Figure 1:**
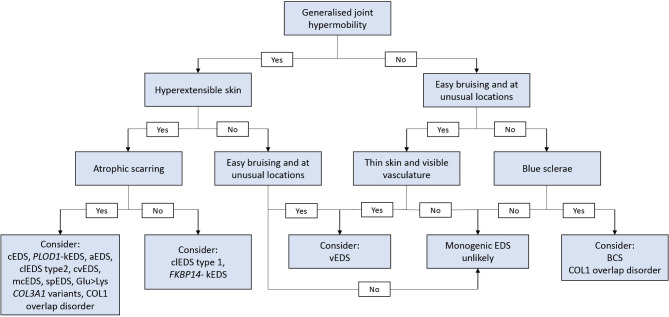
Decision tree to determine likelihood of a diagnosis of a monogenic EDS type in an individual referred with generalised joint hypermobility without a medical or family history with vascular and/or hollow organ rupture.

## 3 Clinical diagnosis of the monogenic EDS types

**Figure 2:**
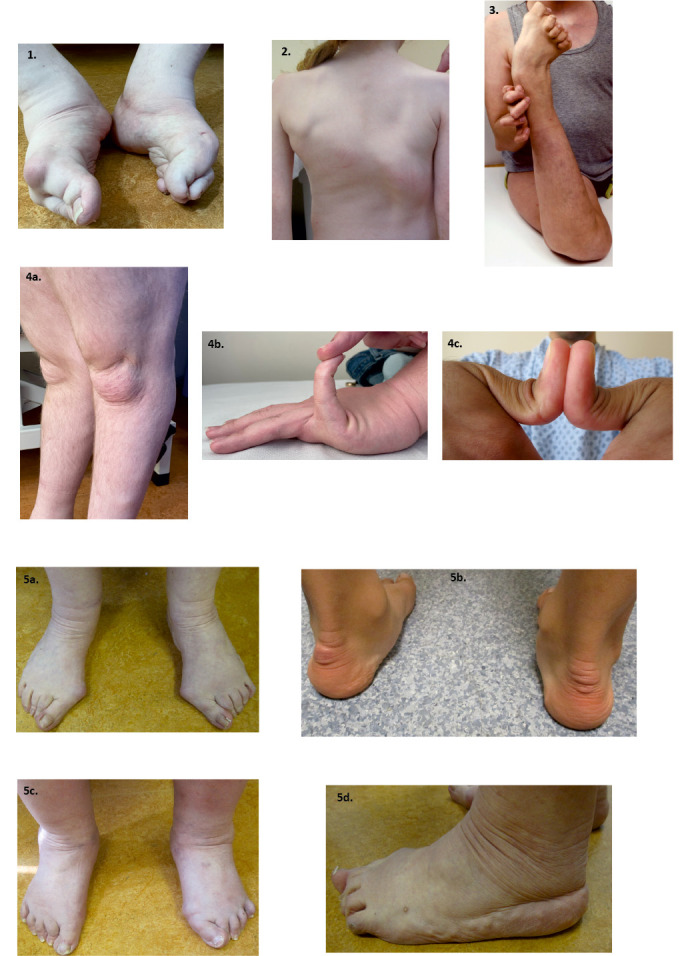
Musculoskeletal features:

Clinical diagnosis starts with a thorough medical history, family history including family tree, and physical examination. To aid the clinical diagnosis of rare EDS types we include a decision diagram (**figure 1**), representing a common scenario of a patient referred with generalised joint hypermobility defined as Beighton score of ≥5 and subsequent clinical assessment for a monogenic type of EDS. This decision tree can be used for streamlining patients into the correct clinical setting depending on the likelihood of a monogenic EDS type. We have also included clinical pictures with appropriate consent in **figure 2A-D** of major and minor characteristics of monogenic EDS types that can be noted on physical examination in individuals with different ethnic backgrounds.

There are several other inherited connective tissue conditions that have overlapping clinical features with the rare EDS types and are important to diagnose as well. These concern for example Loeys-Dietz and Marfan syndromes (arterial aneurysms and blood vessel fragility), Cutis Laxa syndromes (abnormal skin), Osteogenesis Imperfecta (OI) & Stickler syndromes (joint hypermobility) and specific neuromuscular conditions e. g. Bethlem and Ullrich myopathies. Often the genetic causes for these conditions will be present in the requested gene panel. Clotting disorders and non-accidental injury are also in the differential diagnosis because of easy bruising.

## 4 Hypermobile EDS (hEDS) and hypermobility spectrum disorder (HSD)

As mentioned before, hEDS is currently a clinical diagnosis for which specific stringent clinical criteria have been developed in 2017 [5], contrary to previously broad criteria. It has been decided that people with a historical diagnosis of hEDS (criteria: generalised joint hypermobility (Beighton score ≥ 5) and hyperextensible and/or smooth, velvety skin [2]) should retain their diagnosis. The 2017 criteria for hEDS are focussed on adults; for children the 2017 diagnostic criteria for hEDS are not suitable [38]. In our clinical experience it is quite rare for adults to fulfil the 2017 hEDS diagnostic criteria when strictly applied. It is therefore always worth revisiting the clinical features especially if there is mild skin hyperextensibility, and mild atrophic scarring, and assess for COL1 related overlap disorder, or a related inherited connective tissue condition as mentioned above. The HEDGE study aims to arrange whole genome sequencing in 1000 adult individuals fulfilling the 2017 criteria for hEDS but it is considered that most individuals diagnosed with hEDS will not have a monogenic cause and hEDS could represent a multifactorial aetiology.

1. Treatment-resistant talipes in an adult with classical EDS

2. Early-onset (kypho)scoliosis in a child with *FKBP14*-kEDS **(39)**

3. Congenital hip dislocation in an adult with aEDS

4a. Hyperextension of the knee joint >10° in an adult with cEDS

4b. Hypermobiliy of MCP V >90° and DIP V in an adult with cEDS

4c Hypermobility of PIP I in an adult with mEDS

5a. Bilateral hallux valgus in an adult with cEDS

5b. Pes planus and hindfoot deformation in a child with cEDS. Of note, remnants of recurrent heel bursae are visible.

5c. Bilateral broad forefoot and brachydactyly in an adult with *TNXB*- clEDS

5d. Flat foot with multiple piezogenic papules in an adult with *TNXB-*clEDS

**Figure 3:**
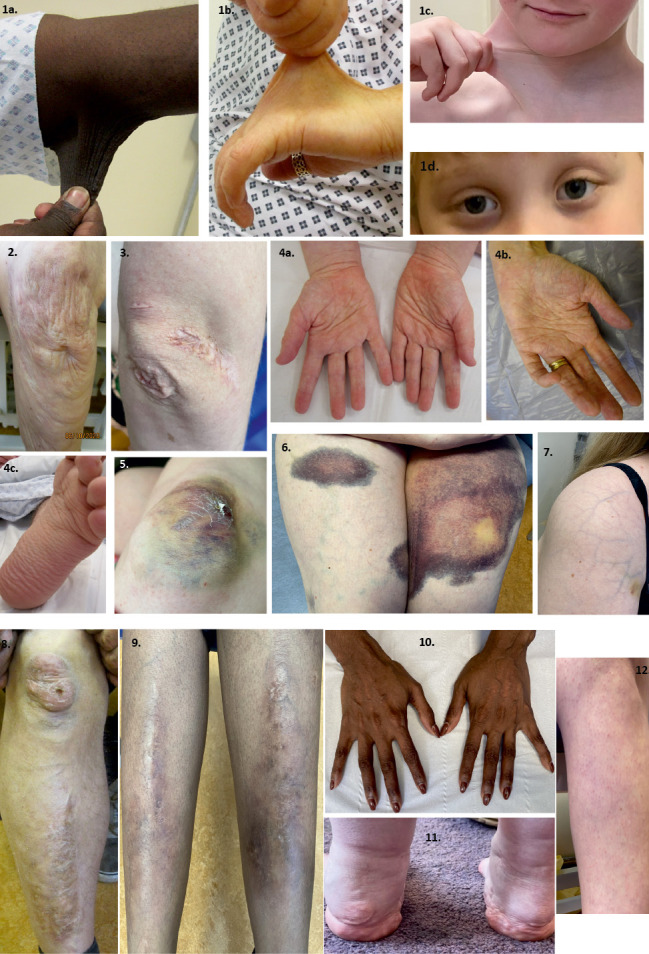
Skin features

1a. Skin hyperextensibility ≥3.0 cm measured at the elbow in an adult with cEDS

1b. Skin hyperextensibility ≥1.5 cm measured at the dorsum of the hand in an adult with *TNXB* -clEDS.

1c. Skin Hyperextensibility ≥3.0 cm of the neck of a child with cEDS.

1d. Epicanthal folds in a child with cEDS.

2. Skin laxity of the skin on the upper leg in an adult with dEDS

3. Small atrophic scars on the elbow of an adult with cEDS

4a. Wrinkling of palms in a child with *FKBP14*- kEDS

4b. Wrinkling of palms in an adult with *TNXB*-clEDS and mallet finger

4c. Wrinkling of soles in an adult with aEDS

5. Recurrent haematoma on the knee of an adult with cEDS

6. Translucent skin with visible veins on upper legs and spontaneous extensive haematoma right and left upper leg in an adult with vascular EDS

7. Translucent skin with visible veins on shoulder of an adult with vascular EDS

8. Molluscoid pseudotumors on the knee and and pretibial haemosiderin deposition in an adult with cEDS

9. Bilateral symmetrical pretibial skin plaques and thin skin in an adult with pEDS

10. Acrogeria of the hands in an adult with pEDS

11. Large piezogenic papules in an adult with a clinical diagnosis of clEDS and a high BMI.

12. Hyperkeratosis follicularis on the shin of an individual with spEDS

**Figure 4:**
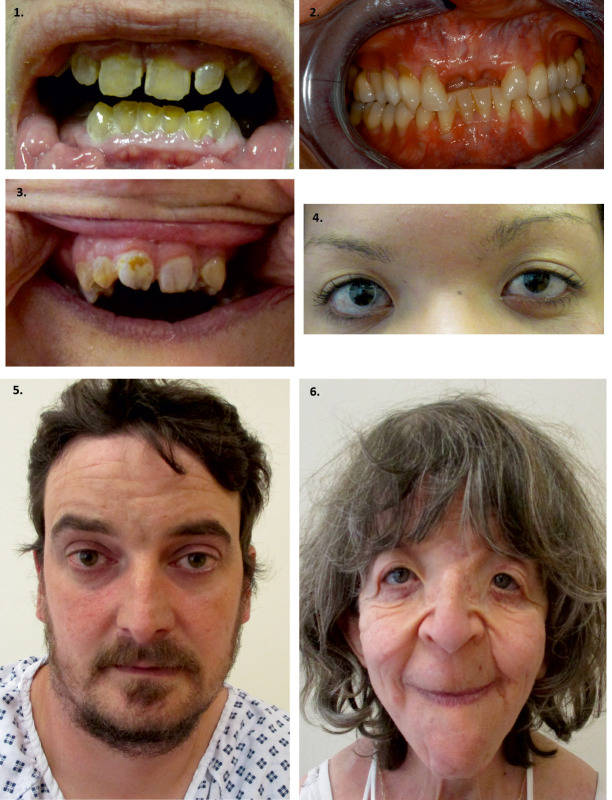
** Other clinical features**:

1. Dentinogenesis imperfecta in an adult with aEDS.

2. Gum recession with generalised lack of attached gingiva in an adult with pEDS

3. Tooth discoloration, dysplasia, and gingival hypertrophy in an adult with dEDS

4. Grey sclerae in an adult with *PLOD1*- kEDS.

5. Facial features of an adult with pEDS which can also be seen in individuals with vEDS including prominent eyes, narrow nose, and thin vermillion of the lips **(33).**

6. Distinctive craniofacial features in an adult with dEDS comprising of hyperterlorism, excessive periorbital skin, epicanthal folds, long and smooth philtrum and chin post implant because of small chin.

The criteria for HSD are broader and will be applicable to many individuals. It is therefore worth emphasizing that the management in people with hEDS and HSD should, because of the variability of clinical problems that can be present in addition to clinical criteria, focus on the individual’s symptoms rather than the diagnosis of hEDS/HSD.

## 5 Conclusion

Correct, precise and extensive description of clinical features (phenotyping) remains an important part of clinical genetic investigations to guide the decision whether to arrange genetic testing and if so, for which conditions. This also applies to the Ehlers-Danlos syndromes. In addition, in those cases where no molecular diagnosis is possible, a clinical diagnosis can still guide management and surveillance and emphasizes the need for additional approaches to identify/confirm a genetic cause.

## Supplementary Material

Supplementary Material

## Notes

We wish to thank the reviewers of the original manuscript for the helpful constructive comments. The Medical University Innsbruck is an Affiliated Partner of the European Reference Network ERN ReCONNET.

## References

[1] Parapia LA, Jackson C. (2008). Ehlers-Danlos syndrome – a historical review. Br J Haematol.

[2] Beighton P, De Paepe A, Steinmann B, Tsipouras P, Wenstrup RJ (1998). Ehlers-Danlos syndromes: revised nosology, Villefranche, 1997. Am J Med Genet.

[3] Malfait F, Castori M, Francomano CA, Giunta C, Kosho T, Byers PH (2020). The Ehlers-Danlos syndromes. Nat Rev Dis Primers.

[4] van Dijk FS, Ghali N, Chandratheva A (2024). Ehlers-Danlos syndromes: importance of defining the type. Pract Neurol.

[5] Richards S, Aziz N, Bale S (2015 May). Standards and guidelines for the interpretation of sequence variants: a joint consensus recommendation of the American College of Medical Genetics and Genomics and the Association for Molecular Pathology. Genet Med.

[6] Malfait F, Francomano C, Byers P (2017). The 2017 international classification of the Ehlers-Danlos syndromes. Am J Med Genet C Semin Med Genet.

[7] Byers PH (2001). Disorders of collagen biosynthesis and structure. The Metabolic and Molecular Bases of Inherited Disease.

[8] Malfait F, Symoens S, Syx D (2024). Classic Ehlers-Danlos Syndrome. GeneReviews®.

[9] Angwin C, Brady AF, Pope FM (2020). Arterial complications in classical Ehlers-Danlos syndrome: a case series. J Med Genet.

[10] Ghali N, Baker D, Brady AF (2019 Sep). Atypical COL3A1 variants (glutamic acid to lysine) cause vascular Ehlers-Danlos syndrome with a consistent phenotype of tissue fragility and skin hyperextensibility. Genet Med.

[11] Byers PH Vascular Ehlers-Danlos Syndrome. GeneReviews®.

[12] Frank M, Adham S, Seigle S (2019 Apr 23). Vascular Ehlers-Danlos Syndrome: Long-Term Observational Study. J Am Coll Cardiol.

[13] Bowen JM, Hernandez M, Johnson DS (2023 Jul). Diagnosis and management of vascular Ehlers-Danlos syndrome: Experience of the UK national diagnostic service, Sheffield. Eur J Hum Genet.

[14] Ghali N, Sobey G, Burrows N (2019). Ehlers-Danlos syndromes. BMJ.

[15] Murray ML, Pepin M, Peterson S, Byers PH (2014). Pregnancy-related deaths and complications in women with vascular Ehlers-Danlos syndrome. Genet Med.

[16] Rohrbach M, Giunta C (2024). PLOD1-Related Kyphoscoliotic Ehlers-Danlos Syndrome. GeneReviews®.

[17] Brady AF, Demirdas S, Fournel-Gigleux S (2017 Mar). The Ehlers-Danlos syndromes, rare types. Am J Med Genet C Semin Med Genet.

[18] Giunta C, Rohrbach M, Fauth C, Baumann M (2019). FKBP14 Kyphoscoliotic Ehlers-Danlos Syndrome. GeneReviews®.

[19] Ayoub S, Ghali N, Angwin C (2020). Clinical features, molecular results, and management of 12 individuals with the rare arthrochalasia Ehlers-Danlos syndrome. Am J Med Genet A.

[20] Martín-Martín M, Cortés-Martín J, Tovar-Gálvez MI (2022). Ehlers-Danlos Syndrome Type Arthrochalasia: A Systematic Review. Int J Environ Res Public Health.

[21] Dhooge T, Van Damme T, Syx D (2021). More than meets the eye: Expanding and reviewing the clinical and mutational spectrum of brittle cornea syndrome. Hum Mutat.

[22] Guarnieri V, Morlino S, Di Stolfo G, Mastroianno S, Mazza T, Castori M (2019). Cardiac valvular Ehlers-Danlos syndrome is a well-defined condition due to recessive null variants in COL1A2. Am J Med Genet A.

[23] Demirdas S, Dulfer E, Robert L (2017 Mar). Recognizing the tenascin-X deficient type of Ehlers-Danlos syndrome: a cross-sectional study in 17 patients. Clin Genet.

[24] Green C, Ghali N, Akilapa R (2020). Classical-like Ehlers-Danlossyndrome: a clinical description of 20 newly identified individuals with evidence of tissue fragility. Genet Med.

[25] van Dijk FS, Ghali N, Demirdas S, Baker D (2022). TNXB-Related Classical-Like Ehlers-Danlos Syndrome. GeneReviews.

[26] Yamaguchi T, Yamada K, Nagai S (2023 Aug 30). Clinical and molecular delineation of classical-like Ehlers-Danlos syndrome through a comprehensive next-generation sequencing-based screening system. Front Genet.

[27] Angwin C, Ghali N, van Dijk FS (2023). Case report: Two individuals with AEBP1-related classical-like EDS: Further clinical characterisation and description of novel AEBP1 variants. Front Genet.

[28] Zong YH, Chijiwa C, Lewis S (2024). Clinical and Molecular Characterization of a Novel Homozygous Frameshift Variant in AEBP1-Related Classical-like Ehlers Danlos Syndrome Type 2 with Comparison to Previously Reported Rare Cases. Genes (Basel).

[29] Yamaguchi T, Hayashi S, Nagai S (2023 May 5). Case report: further delineation of AEBP1-related Ehlers-Danlos Syndrome (classical-like EDS type 2) in an additional patient and comprehensive clinical and molecular review of the literature. Front Genet.

[30] Furuhata-Yoshimura M, Yamaguchi T, Izu Y, Kosho T (2023). Homozygous splice site variant affecting the first von Willebrand factor A domain of COL12A1 in a patient with myopathic Ehlers-Danlos syndrome. Case Reports Am J Med Genet A.

[31] Kapferer-Seebacher I, van Dijk FS, Zschocke J (2021 Jul 29). Periodontal Ehlers-Danlos Syndrome. GeneReviews®.

[32] Kapferer-Seebacher I, Oakley-Hannibal E, Lepperdinger U (2021 Feb). Prospective clinical investigations of children with periodontal Ehlers-Danlos syndrome identify generalized lack of attached gingiva as a pathognomonic feature. Genet Med.

[33] Angwin C, Zschocke J, Kammin T (2023 May 31). Non-oral manifestations in adults with a clinical and molecularly confirmed diagnosis of periodontal Ehlers-Danlos syndrome. Front Genet.

[34] Minatogawa M, Unzaki A, Morisaki H (2022 Sep). Clinical and molecular features of 66 patients with musculocontractural Ehlers-Danlos syndrome caused by pathogenic variants in CHST14 (mcEDS-CHST14). J Med Genet.

[35] Minatogawa M, Hirose T, Mizumoto S (2022). Clinical and pathophysiological delineation of musculocontractural Ehlers—Danlos syndrome caused by dermatan sulfate epimerase deficiency (mcEDS-DSE): A detailed and comprehensive glycobiological and pathological investigation in a novel patient. Hum Mutat.

[36] Morlino S, Micale L, Ritelli M (2020). COL1-related overlap disorder: A novel connective tissue disorder incorporating the osteogenesis imperfecta/Ehlers-Danlos syndrome overlap. Clin Genet.

[37] Venable E, Knight DRT, Thoreson EK, Baudhuin LM (2023 Jun). COL1A1 and COL1A2 variants in Ehlers-Danlos syndrome phenotypes and COL1-related overlap disorder. Am J Med Genet C Semin Med Genet.

[38] Tofts LJ, Simmonds J, Schwartz SB, Richheimer RM, O’Connor C, Elias E, Engelbert R, Cleary K, Tinkle BT, Kline AD, Hakim AJ, van Rossum MAJ, Pacey V (2023). Pediatric joint hypermobility: a diagnostic framework and narrative review. Orphanet J Rare Dis.

[39] Giunta C, Baumann M, Fauth C (2018). A cohort of 17 patients with kyphoscoliotic Ehlers-Danlos syndrome caused by biallelic mutations in FKBP14: expansion of the clinical and mutational spectrum and description of the natural history. Genet Med.

