# Supplementary material for: Clinical diagnosis of the monogenic Ehlers-Danlos syndromes
Source: Med Genet. 2024 Dec 3;36(4):225–34. doi: 10.1515/medgen-2024-2060 (PMC11610443; doi:10.1515/medgen-2024-2060)
Supplement: Supplementary file 1 — Supplementary Material [file SupplementaryTable1.docx]

**Supplementary Table 1. Gene specific criteria for kEDS & spEDS.**

| **kEDS** | **spEDS** |
| --- | --- |

| *PLOD1* | *FKBP14* | *B4GALT7* | *B3GALT6* | *SLC39A3* |
| --- | --- | --- | --- | --- |
| 1.Skin fragility (easy bruising, friable skin, poor wound healing), widened atrophic scarring  2.Scleral and ocular fragility/rupture  3.Microcornea  4.Facial dysmorphology | 1.Congenital hearing impairment (sensorineural, conductive, or mixed)  2.Follicular hyperkeratosis  3.Muscle atrophy  4.Bladder diverticula | 1. Radioulnar synostosis  2. Bilateral elbow contractures or limited elbow movement  3. Generalized joint hypermobility  4. Single transverse palmar crease  5. Characteristic craniofacial features  6. Characteristic radiographic findings  7. Severe hypermetropia  8. Clouded cornea | 1. Kyphoscoliosis (congenital or early onset, progressive)  2. Joint hypermobility, generalized or constricted to distal joints, with joint dislocations  3. Joint contractures (congenital or early onset, progressive) (especially hands)  4. Peculiar fingers (slender, tapered, arachnodactyly, spatulate, with broad distal phalanges)  5. Talipes equinovarus  6. Characteristic craniofacial features  7. Tooth discoloration, dysplastic teeth  8. Characteristic radiographic findings  9. Osteoporosis with multiple spontaneous fractures  10. Ascending aortic aneurysm  11. Lung hypoplasia, restrictive lung disease | 1.Protuberant eyes with bluish sclerae  2. Hands with finely wrinkled palms  3. Atrophy of the thenar muscles and tapering fingers  4. Hypermobility of distal joints  5. Characteristic radiologic findings |
